# An Efficient Stepwise Statistical Test to Identify Multiple Linked Human Genetic Variants Associated with Specific Phenotypic Traits

**DOI:** 10.1371/journal.pone.0138700

**Published:** 2015-09-25

**Authors:** Iksoo Huh, Min-Seok Kwon, Taesung Park

**Affiliations:** 1 Department of Statistics, Seoul National University, Gwanak-gu, Seoul, Korea; 2 Interdisciplinary Program in Bioinformatics, Seoul National University, Gwanak-gu, Seoul, Korea; Indiana University Bloomington, UNITED STATES

## Abstract

Recent advances in genotyping methodologies have allowed genome-wide association studies (GWAS) to accurately identify genetic variants that associate with common or pathological complex traits. Although most GWAS have focused on associations with single genetic variants, joint identification of multiple genetic variants, and how they interact, is essential for understanding the genetic architecture of complex phenotypic traits. Here, we propose an efficient stepwise method based on the Cochran-Mantel-Haenszel test (for stratified categorical data) to identify causal joint multiple genetic variants in GWAS. This method combines the CMH statistic with a stepwise procedure to detect multiple genetic variants associated with specific categorical traits, using a series of associated I × J contingency tables and a null hypothesis of no phenotype association. Through a new stratification scheme based on the sum of minor allele count criteria, we make the method more feasible for GWAS data having sample sizes of several thousands. We also examine the properties of the proposed stepwise method via simulation studies, and show that the stepwise CMH test performs better than other existing methods (e.g., logistic regression and detection of associations by Markov blanket) for identifying multiple genetic variants. Finally, we apply the proposed approach to two genomic sequencing datasets to detect linked genetic variants associated with bipolar disorder and obesity, respectively.

## Introduction

Many comprehensive genome-wide association studies (GWAS) have now been conducted to identify previously unknown single nucleotide polymorphisms (SNPs) associated with numerous normal and pathological phenotypes. These novel genetic markers are well tabulated in GWAS catalogs that are updated regularly [1]. However, the majority of such genetic markers are obtained via single-marker analysis, due to the constraints of commonly used statistical methods for judging genetic associations [2,3]. This limitation is problematic to the advancement of biomedicine, as it is now known that complex diseases which severely impact the health of the general population, are coordinately influenced by multiple genetic factors.

Because the biological and biochemical pathways related to genetic markers often interact to induce disease states, the importance of identifying specific pathway-associated genetic variants is increasingly being recognized [4,5]. Furthermore, joint identification may improve the predictive performance of specific types of statistical analyses [6]. However, traditional models, with logistic regression as one salient example, cannot easily handle multiple genetic markers [4,7,8] when there are sparse cell counts in contingency tables of a categorical trait and single nucleotide polymorphism (SNP) combinations, because the standard errors of parameter estimates tend to be inflated, and the p-value becomes close to one. In addition, extensive computing time is required for estimating parameters in the presence of numerous SNPs.

Consequently, several methods have been proposed to overcome these problems. One such method, penalized logistic regression, is efficient when a large set of SNPs is needed [6]. For example, when penalized logistic regression is fitted, multiple SNPs expected to jointly affect a disease phenotype are selected from the model. However, this approach is not able to simultaneously handle whole SNP datasets owing to its intensive computational burden. Therefore, a small number of SNPs (e.g., 1000 SNPs), having strong marginal effects, are filtered from a single SNP analysis prior to the application of penalized logistic regression [6]. Even with this limitation, in most cases, penalized logistic regression still provides a large number of SNPs, which complicates further biological interpretation.

A second approach, multifactor dimensionality reduction (MDR), is a nonparametric, model-free, and combinatorial approach for interaction analysis that identifies a multi-locus model for association in case-control studies [9]. The MDR method reduces multi-locus genotypes into high- vs. low-risk disease groups. If the ratio of cases and controls in a combination of genotypes is larger than a pre-assigned threshold T (e.g., T = 1), the cell of combination is labeled “high risk;” if smaller than the threshold, it is labeled “low risk.” Based on the label of each cell in the contingency table, MDR runs 10-fold cross-validation to select an SNP set with the smallest prediction error and/or the most consistently large training accuracies. Thus, this method avoids the sparsity problem by assuming that sparse cells are undefined. However, since MDR selects k-way interactions purely by the prediction performance of an exhaustive search, it is impractical to detect high-order interactions. Additionally, although some MDR approaches were proposed to reduce computation time [10], or space searching [11], detection of > 3-way interactions from GWAS data is not yet possible.

In addition to the above, a variety of other methods have been proposed to identify gene-gene interactions. For example, Detection of ASSOciations using Markov Blanket (DASSO-MB) was proposed to detect interactions via a Markov blanket used to shield a specific variable from all other variables [12]. This method employs a goodness-of-fit test combined with a stepwise procedure. In some simulation settings, this method outperforms MDR. However, the method has their own drawbacks, such as increased degrees of freedom upon the addition of SNPs.

In this report, we propose a stepwise method for the identification of SNPs that jointly associate with specific phenotypes. Our method uses Cochran-Mantel-Haenszel (CMH) statistics, commonly used in contingency table analysis [13,14] to sequentially test the conditional independence phenotypes from genetic factors. Although the use of CMH statistics for association tests was previously proposed [15], it implementation has proved impractical to handle GWAS datasets, due to the number of strata, derived by distinct genotype combinations, increasing exponentially with increased size of the selected SNP set. To resolve this limitation, we propose a new criterion for stratification categorized by the sum of minor allele counts (MACs). This categorization alleviates intensive computational burden, and therefore facilitates the joint identification of high-order SNPs in large sample datasets. In addition, we also discuss application of ordinal phenotypes. In the results section, we use simulations to compare our method with stepwise logistic regression and DASSO-MB. Finally, we apply our modified CMH approach to two GWAS datasets to detect collective multiple genetic variants related to bipolar disorder and obesity, respectively.

## Materials and Methods

### Generalized CMH Method

The original CMH test proposed by Mantel and Haenszel is the method to tests conditional independence of 2×2×*K* contingency tables [16,17], meaning that the method is commonly used to test for conditional independence between two binary variables, after adjusting for the effect of confounding variables with *K* strata. Statistics of the test follow chi-square distributions with one degree of freedom and perform best when the associations of two binary variables have the same directions in each partial table. Situational application of this approach has been generalized by Birch [18], Landis [19], and Mantel [20] to an *I*×*J*×*K* table in which the predictor variable and the response variable have *I* and *J* levels, respectively, that can be treated as not only nominal but also as ordinal. Therefore, the generalized CMH method consists of two more tests, in addition to the conditional independence test for two nominal variables. One test examines the mean score difference when one variable is ordinal, and the other test evaluates the correlation when both variables are ordinal [20]. The generalized CMH statistics is given as

$$L^{2}=[\sum_{k}B_{k}(n_{k}-\mu_{k})]'{\left[\sum_{k}B_{k}V_{k}B_{k}^{'}\right]}^{-1}[\sum_{k}B_{k}(n_{k}-\mu_{k})]$$

In the above equation, ***B***_***k***_ is the Kronecker product between the row score **u**_**k**_ and the column score **v**_**k**_, **n**_**k**_ and **μ**_**k**_ are vectors of observed and expected counts of length of *I*×*J* in the k^th^ strata, respectively. ***V***_***k***_ is an (*I*×*J*)×(*I*×*J*) variance matrix of **n**_**k**_, evaluated under an assumed hypergeometric distribution. Therefore, **n**_**k**_ and **μ**_**k**_ is represented as (*n*_11*k*_,*n*_12*k*_,…,*n*_*IJk*_) and (*n*_1+*k*_×*n*_+1*k*_,*n*_1+*k*_×*n*_+2*k*_,…,*n*_*I*+*k*_×*n*_+*Jk*_)/*n*_++*k*_ respectively. Moreover, elements of ***V***_***k***_ consist of covariance terms between *n*_*ijk*_ and *n*_*i*'*j*'*k*_, and are represented as $n_{i+k}(\omega_{ii'}n_{++k}-n_{i'+k})n_{+jk}(\omega_{jj'}n_{++k}-n_{+j'k})/(n_{++k}^{2}(n_{++k}-1))$ where *ω*_*ab*_ = 1, when *a* = *b* and *ω*_*ab*_ = 0 otherwise.

Three types of tests can be derived by imposing ordinal or nominal weights on **u**_**k**_ and **v**_**k**_. When **u**_**k**_ is used as the nominal variable, it is described as a (*I*−1)×*I* matrix (***I***, **-1)**, where ***I*** is an identity matrix of size *I*−1, and **1** denotes a column vector of *I*−1 ones. When **u**_**k**_ is used as the ordinal variable, it is given as (*u*_1_,*u*_2_,…,*u*_*I*_), with an ordered score vector given to each level of predictor. **v**_**k**_ is constructed similarly with **u**_**k**_. Therefore, the general association test is conducted if both variables are nominal, the mean score test is conducted if only one variable is ordinal, and the correlation test is conducted if both variables are ordinal. The degrees of freedom are given as (*I*−1)×(*J*−1) for the general association test, *I*−1 or *J*−1 for the mean score test, and 1 for the correlation test [21].

### Application of the CMH Method to SNP Data

**Fig 1:**
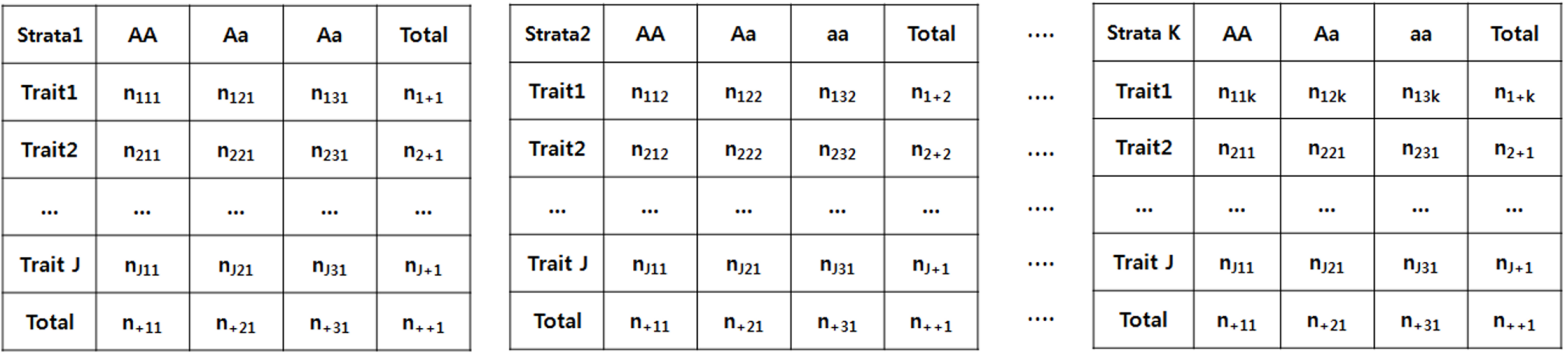
Contingency table for CMH test between trait and *p* SNPs. This test can be represented as H_0_: *Y*⊥SNP|(number of *p*-1 SNPs). “A” and “a” represent major and minor alleles, respectively.

We next applied the CMH test to identify genetic variants that mutually associate with a trait of interest. Let *Y* represent the trait status of a subject, for example, a specific disease. The number of values which *Y* can have is two for a binary trait, or >2 for ordinal or multinomial traits. Let *I* denote the number of values which *Y* can have. If there is one SNP associated with Y, the data can be summarized by an *I*×3 contingency table. We further assume that the genetic model is codominant for generality, and the CMH test is performed without stratification. When there are two SNPs, *S*_1_ and *S*_2_, the data can be summarized in a *I*×3×3 contingency table, and the CMH test evaluates the conditional independence between *Y* and *S*_1_, given *S*_2_. When there are three SNPs, *S*_*1*_, *S*_*2*_, and *S*_*3*_, the data can be summarized in a *I*×3×3^2^ contingency table and the CMH tests the association between *Y* and *S*_*1*_, given (*S*_*2*_, *S*_*3*_). Consequently, the genetic frequency data of case-control study can be summarized in an *I*×*J*×*K* contingency table, where *I* = 2 *J* = 3, *K* = 3^p-1^, and *p* = the total number of SNPs (Fig 1). However, this stratification scheme has the potential problem that *K* would be too large in the case of many SNPs. Because this scheme may require excessive computation, it is impractical to apply this stratification scheme to GWAS data. For example, if there are 10 SNPs in hand, the number of strata could reach 3^9^ = 19683, indicating that this dataset is too divided to accurately reflect its properties. Therefore, we propose an alternative stratification scheme, based on the assumption that subjects with similar sums of minor allele counts (MACs) may have similar risks of disease traits [22,23]. In addition, we can fix the maximum number of strata through clustering subjects whose MACs exceed a predefined criterion as one stratum. This new scheme makes computation much faster than the former scheme, and we expect that several dozen SNPs can be easily identified in a reasonable time.

### Stepwise CMH Test

The general CMH test has (*I*−1)×(*J*−1) degrees of freedom. Therefore, when we apply this test to the codominant genetic model, it follows a chi-squared distribution with (*I*−1)×2 degrees of freedom at most. Next, the test for the additive, dominant or recessive genetic models can be similarly developed using the mean score of the CMH test [20].

To identify multiple causal SNPs among a large total number of SNPs, we propose the following stepwise CMH procedure, following determination of stratification criteria based on MACs. Firstly, in the forward step, the most significant SNP associated with the disease of interest is added to the previously selected SNPs via the CMH test. Therefore, if there are *N* SNPs in the total dataset, and *p* (= 0 for the first step) SNPs previously selected by the test, a SNP whose CMH statistic p-value conditioned on the *p* selected SNPs is the smallest and smaller than the threshold would be added. For the first forward step, the CMH test is applied without stratification.

Secondly, in the backward step, we implement the CMH test to remove the least significant SNPs among the previously selected SNP set. All SNPs in the set are tested in the presence of all of the other SNPs. If any SNP has a p-value that exceeds the removal threshold, the SNP whose p-value is largest is excluded from the SNP set; otherwise, this step does not remove any SNPs. This backward step can be optionally skipped by the researcher.

Our stepwise CMH method iterates between the forward and backward steps until no additional variable is added to the current model at the forward step. Once a set of significant SNPs is identified, these are removed from the whole SNP dataset, and the first step of the stepwise procedure is repeated. The whole stepwise procedure is repeated until no more significant SNPs are selected in the first forward step.

## Results and Discussion

### Simulation Studies

To investigate the utility of our stepwise CMH method, we conducted a simulation study to compare it with the logistic regression and DASSO-MB approaches. For simplicity, we only considered the main effects of two and three causal SNP models. The odds and penetrances for the three causal SNP model are provided in Tables 1 and 2 [24]. The corresponding true logistic model for binary traits is assumed as follows:

$$\mathrm{logit}(p(y=1))=\beta_{0}+\sum_{p=1}^{P}\beta_{p}\times SNP_{p}$$

**Table 1:** Odds table for simulation studies and binary trait in three causal SNP model (*θ*_*0*_ = 0).

((#of c) = *k*)	*AA*	*Aa*	*Aa*
*BB*	α(1+*θ*_*k*_)	α(1+*θ*_*1*_)(1+*θ*_*k*_)	α(1+*θ*_*2*_)(1+*θ*_*k*_)
*Bb*	α(1+*θ*_*1*_)(1+*θ*_*k*_)	α(1+*θ*_1_)^2^(1+*θ*_*k*_)	α(1+*θ*_*1*_)(1+*θ*_*2*_)(1+*θ*_*k*_)
*Bb*	α(1+*θ*_*2*_)(1+*θ*_*k*_)	α(1+*θ*_*1*_)(1+*θ*_*2*_)(1+*θ*_*k*_)	α(1+*θ*_*2*_)^2^(1+*θ*_*k*_)

**Table 2:** Penetrance table for Table 1 in three causal SNP model (*θ*_*0*_ = 0).

(#of *c* = *k*)	*AA*	*Aa*	*Aa*
*BB*	$\frac{\alpha (1+\theta_{k})}{1+\alpha (1+\theta_{k})}$	$\frac{\alpha (1+\theta_{1})(1+\theta_{k})}{1+\alpha (1+\theta_{1})(1+\theta_{k})}$	$\frac{\alpha (1+\theta_{2})(1+\theta_{k})}{1+\alpha (1+\theta_{2})(1+\theta_{k})}$
*Bb*	$\frac{\alpha (1+\theta_{1})(1+\theta_{k})}{1+\alpha (1+\theta_{1})(1+\theta_{k})}$	$\frac{\alpha {\left(1+\theta_{1}\right)}^{2}(1+\theta_{k})}{1+\alpha {\left(1+\theta_{1}\right)}^{2}(1+\theta_{k})}$	$\frac{\alpha (1+\theta_{1})(1+\theta_{2})(1+\theta_{k})}{1+\alpha (1+\theta_{1})(1+\theta_{2})(1+\theta_{k})}$
*bb*	$\frac{\alpha (1+\theta_{2})(1+\theta_{k})}{1+\alpha (1+\theta_{2})(1+\theta_{k})}$	$\frac{\alpha (1+\theta_{1})(1+\theta_{2})(1+\theta_{k})}{1+\alpha (1+\theta_{1})(1+\theta_{2})(1+\theta_{k})}$	$\frac{\alpha {\left(1+\theta_{2}\right)}^{2}(1+\theta_{k})}{1+\alpha {\left(1+\theta_{2}\right)}^{2}(1+\theta_{k})}$

To generate datasets according to the true model, we first determined the total penetrance, which defines the proportion of cases in whole samples. Then, we set the values for the baseline effect α and genetically additive effect θ. In case of the codominant model and binary traits, θ has two values: θ_1_ and θ_2_, where θ_1_ is the marginal effect between major homogeneous and heterogeneous genotypes, given by 0.7, and θ_2_ is the marginal effect between major and minor homogeneous genotypes, given by 0.5.

In this simulation study, we assumed linkage equilibrium between causal SNPs, with minor allele frequencies (MAFs) set to 0.03, 0.05, 0.1, and 0.2. We then generated 1000 datasets, each consisting of 1000 cases, 1000 controls. We set the number of SNPS to be 100, 300, 500, and 1,000 (including non-causal SNPs). Two accuracy measures were used to compare the stepwise CMH method to others. First, the detection probability (Dprob) was estimated by dividing the number of correctly captured SNPs by the total number of true SNPs. Second, the proportion of datasets out of all 1000 datasets that detected all of the causal SNPs was evaluated (power). In addition, two threshold values were used to evaluate significance: Bonferroni correction criterion 5×10^−4^ and a looser criterion 5×10^−3^.

**Fig 2:**
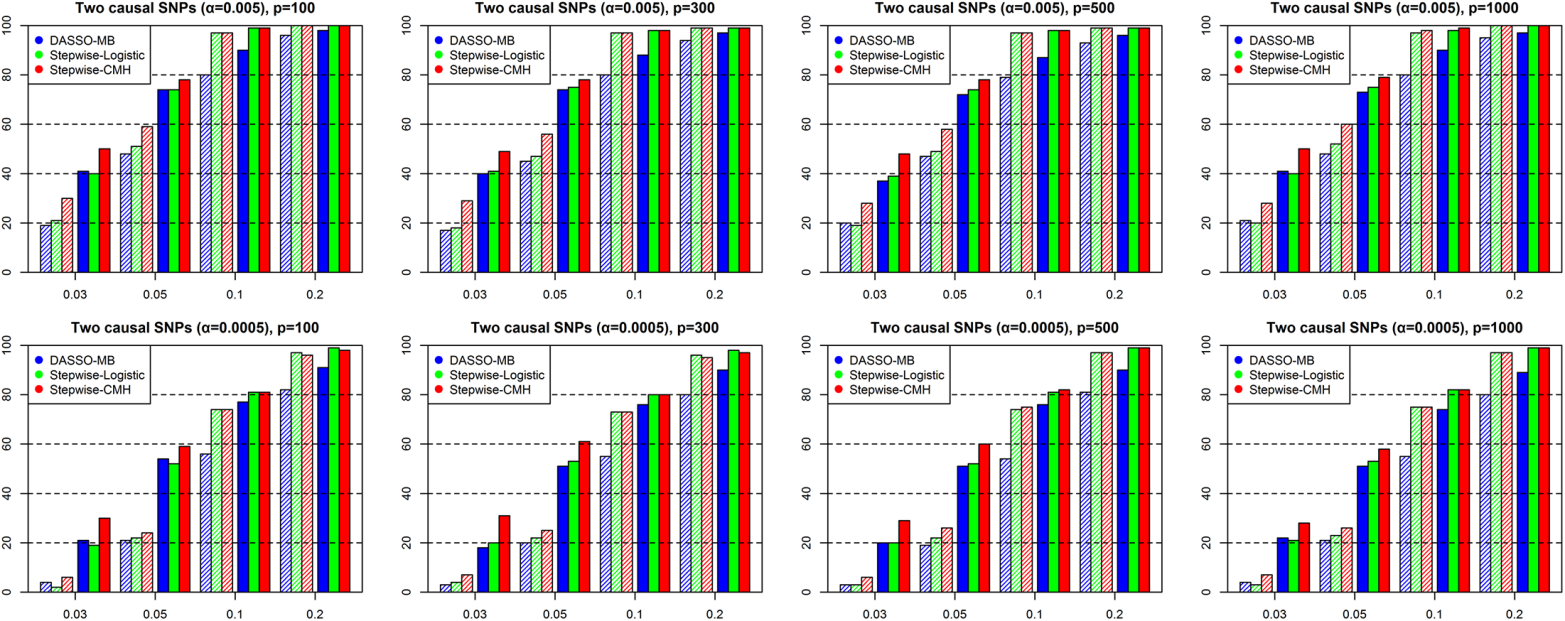
Performance comparison of stepwise CMH method, stepwise logistic and DASSO-MB methods for the codominant model with two causal SNPs. Blue bars represent the result of the DASSO-MB method, green bars represent the result of the stepwise logistic method, and red bars represent the result of the stepwise CMH method. Bars with diagonal lines are the results of power and solid bars are those of Dprob. The x-axis represents the MAF of true causal SNPs and the y-axis represents the value of two accuracy measures based on three approaches.

**Fig 3:**
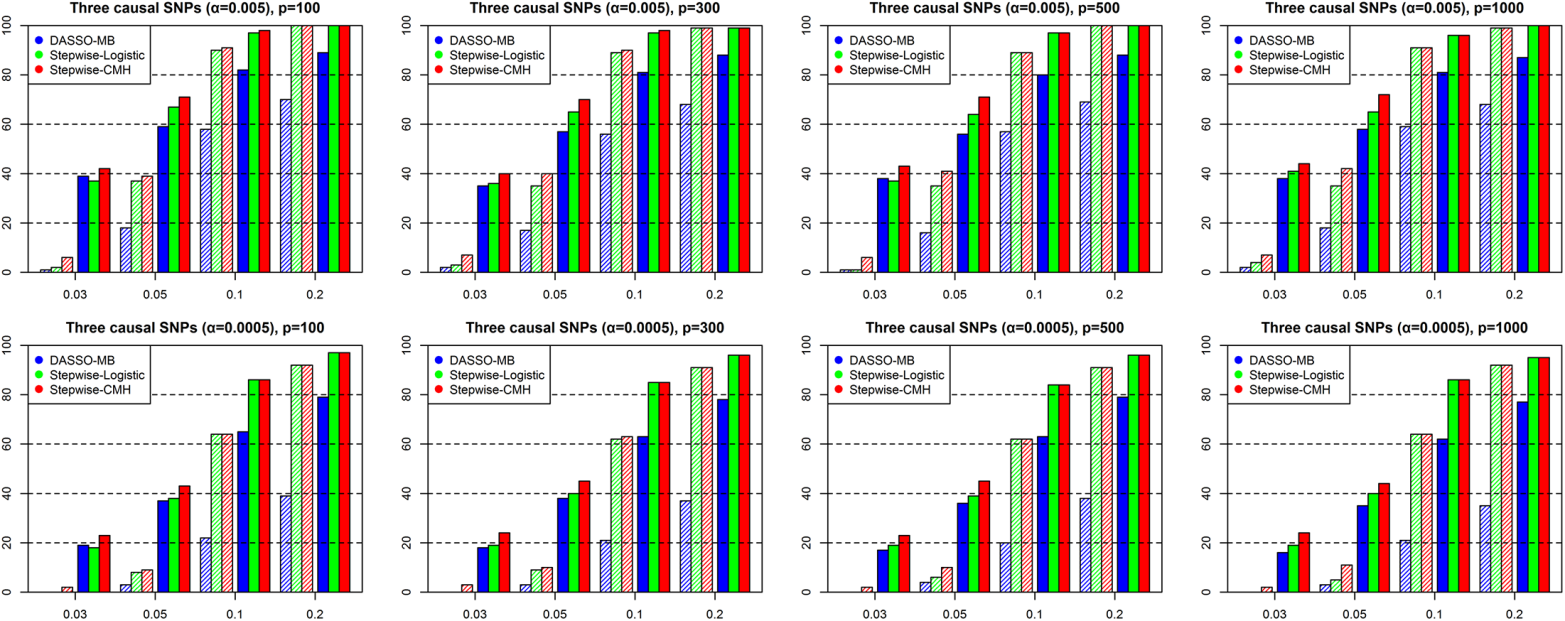
Performance comparison of stepwise CMH method, stepwise logistic and DASSO-MB methods for the codominant model with three causal SNPs.

The simulation results are shown in Figs 2 and 3. Fig 2 shows the results for the model with two causal SNPs, and Fig 3 for the model with three causal SNPs. For the codominant model with binary traits, both accuracy measures for the stepwise CMH method, when the MAF was relatively low (0.03 and 0.05), were clearly greater than those of the stepwise logistic method. However, with moderate MAFs (0.10 and 0.20), the two approaches provided comparable results. This is because when the MAF value is small enough to induce sparse minor allele counts of some strata, logistic regression produces very large standard errors, and some p-values of the coefficients are greatly inflated. However, the CMH statistic has a more robust variance estimate that is not substantially affected by sparse cells, due to the fact that the CMH variance is only calculated using marginal counts. Therefore, the performance of the stepwise CMH method is better than that of the stepwise logistic method. This pattern is consistent regardless of the number of SNPs.

By contrast, DASSO-MB generally showed low power and Dprob values, with a few exceptions. DASSO-MB is unfavorable in situations when a model contains only main effects, due to increased degrees of freedom of tests with increased numbers of variables included in the selected set. For example, if the second SNP in the forward step was tested once a SNP was already selected, the degrees of freedom could reach six (Fig 2), while including a third variable could result in 18 degrees of freedom (Fig 3). Such increases in degrees of freedom result in decreased power.

### Comparison of CMH with Two Other Methods via a Toy Example

**Fig 4:**
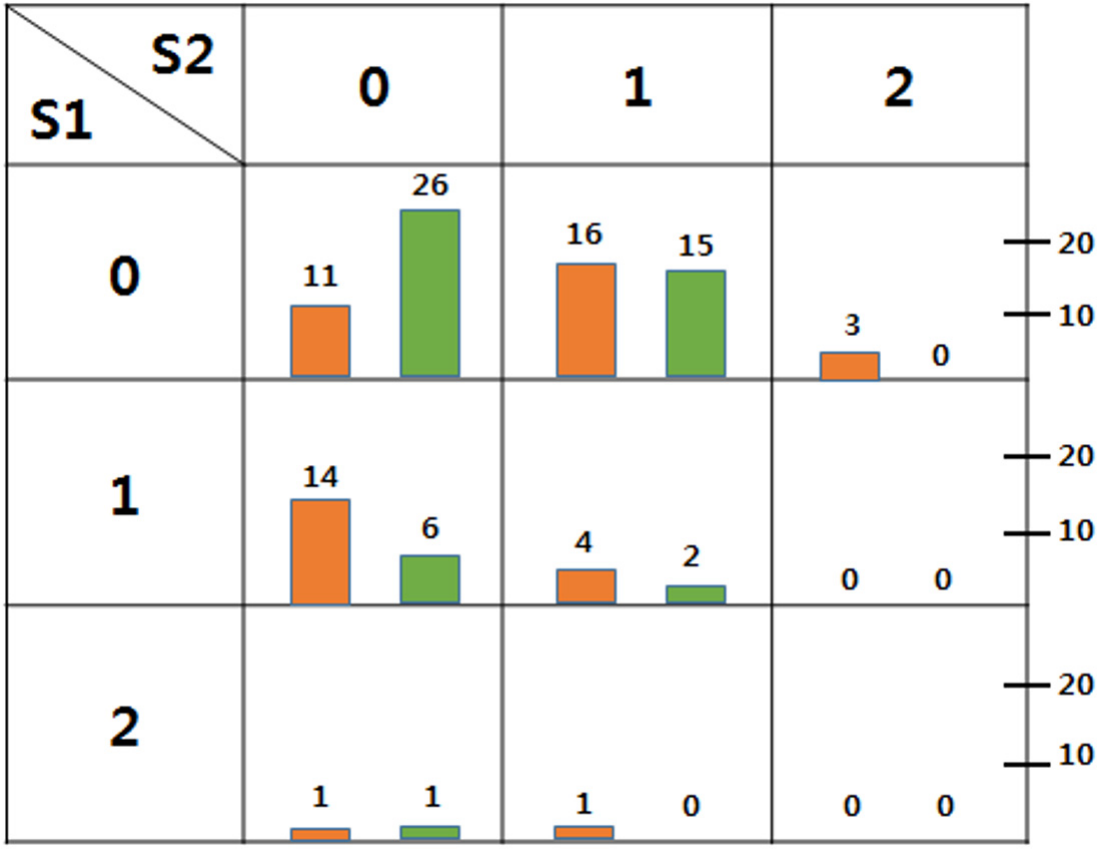
Toy example dataset structure to show superiority of the stepwise CMH method. We counted the numbers of cases and controls for each genotype combination and expressed them as vertical bars to visualize. Orange bars represent the counts of cases and the green bars do counts of controls.

To demonstrate the superiority of the stepwise CMH method more clearly, we provided an artificially generated toy example consisting of samples of 50 cases and 50 controls, and genotypes of two informative SNPs denoted *S*_*1*_ and *S*_*2*_, respectively. The structure of the dataset is shown in Fig 4. If we set an entrance cutoff of 0.05, *S*_*1*_ would be selected in the first forward step by all three methods, because all three p-values would be < 0.05. However, in the second forward step, *S*_*2*_ was selected only by the CMH method, while some sparse cells in the genotype table resulted in p-value inflation in logistic regression (Fig 4, Table 3). DASSO-MB also could not detect the second SNP, because this method allows larger degrees of freedom than the other two methods (Table 3).

**Table 3:** Toy example dataset application result.

Methods	p-value (1^st^ forward step)	DF	p-value (2^nd^ forward step)	DF
Stepwise CMH	0.0146	2	0.0304	2
Stepwise Logistic	0.0203	2	0.2450	2
DASSO-MB	0.0498	2	0.1330	6

### Application of the CMH Approach for Analyzing Wellcome Trust Case Control Consortium (WTCCC) Bipolar Disorder Data

We next applied our proposed CMH method to a WTCCC bipolar disorder (BD) dataset. BD is well known to be highly heritable and polygenic [25,26], and the dataset consisted of 2,938 controls and 1,868 cases, and a total of 354,022 genome-wide SNP markers. We filtered out SNP markers with MAFs < 1%, or having call rates < 95%. We also selected tagged SNPs, based on the criteria of r^2^ > 0.5 between 50 adjacent SNPs. These filtering steps resulted in 134,254 SNPs usable for our analysis. We then applied the stepwise CMH method to the dataset, based on a codominant genetic model. We set the entry and removal threshold value of significance as *α* = 5×10^−5^. After all the stepwise procedures were completed, two SNP sets were selected (Table 4).

**Table 4:** WTCCC bipolar disorder data analysis result (Entrance cutoff = 5×10^−5^, Removal cutoff = 5×10^−5^).

Set	SNP name	GENE
1^st^ Set	*rs11112069*,*rs420259*,*rs17484671*, *rs7260296*,*rs4918068*,*rs6705537*, *rs12594576*, *rs6908950*, *rs11984645*, *rs8021692*, *rs4027132*, *rs4276227*, *rs7152966*, *rs17561681*, *rs9510385*, *rs4567706*	*CHST11*, *PALB2*, *NR3C2*, *PNPLA6*, *OBFC1*, *USP34*, *FAH*, *GLTSCR1L*, *OPRK1*, *TDRD9*, *LPIN1*, *CMTM8*, *TSHR*, *GABRA5*, *unknown*, *unknown*,
2^nd^ Set	*rs7184080*, *rs12537100*, *rs2609653*	*LOC101928392*, *THSD7A*, *unknown*,

In the first SNP set, *rs11112069*, located in the gene *CHST11*, encoding carbohydrate sulfotransferase-11, which catalyzes sulfate transfer to position 4 of the N-acetylgalactosamine (GalNAc) residue of chondroitin. Chondroitin sulfate is known to facilitate axonal patterning and cell migration during the early growth and development of the mammalian central nervous system [27]. *CHST11* is also related to neuronal function, suggesting a possible (but yet unknown) relationship to BD. Another SNP, *rs420259*, is located in the partner and localizer of *BRCA2* (*PALB2*), which facilitates DNA repair by recruiting *BRCA2* and *RAD51* to double-stranded DNA breaks, and *PALB2* and *BRCA2* have been associated with both BD and schizophrenia in a Scandinavian study [28]. *BRCA2*, expressed in the mouse brain, was shown to be important for normal neurogenesis, particularly in the cerebellum, a region involved in emotional processing that is often dysfunctional in BD [29]. *rs17484671* is located in the gene NR3C2, encoding nuclear receptor subfamily 3, group C, and member 2 [30], the drug target for dipolar disorder [31]. The SNP *rs12537100* is located in an intronic region of the gene *THSD7A*, thrombospondin, type I, domain containing 7A. Thrombospondins are key regulators of synaptogenesis in the central nervous system [32]. *rs7260296*, located 10KB downstream of the gene *NTE*, also known as *PNPLA6*, patatin-like phospholipase domain containing 6. NTE is a lysophospholipase that maintains intracellular phospholipid homeostasis by converting lysophosphatidylcholine to glycerophosphocholine [33]. *PNPLA6* is directly related to neuronal function [34], and its dysfunction may associate with the onset of BD. Moreover, the majority of other SNPs in our set were reported previously [35–38].

In summary, many selected SNPs directly or indirectly related to neuronal function. Therefore, joint identification of the putative causal SNPs could provide more biologically meaningful interpretation and motivation of further investigation, such as pathway analysis.

### Application to Korea Association Resource (KARE) project

We also applied our stepwise CMH method to a GWA dataset from the Korean Association REsource (KARE) project, initiated in 2007 to undertake a large-scale GWAS of 260 traits among 10,038 participants (aged between 40 and 69) of Ansung (n = 5,018) and Ansan (n = 5,020) population-based cohorts. Among the 260 traits, we selected body mass index (BMI) to detect causal variants associated with obesity. Here, BMI was treated as an ordinal variable with four categories: normal (18.5 ≤ BMI < 25), overweight (25 ≤ BMI < 30), mildly obese (30 ≤ BMI < 35), and severely obese (35 ≤ BMI < 40), and the subjects were numbered from 1 (normal) to 4 (severe), respectively. The dataset consisted of 8842 individuals with a total of 352,228 genome-wide SNPs. We filtered out SNP markers with MAFs < 1% or call rates < 95%. We also selected tagged SNPs, based on the criteria of r^2^ > 0.5 between 50 adjacent SNPs. These criterions resulted in 137,400 SNPs usable for our analysis, with the same cutoff used in our analysis of the BD dataset. After all stepwise procedures were completed, the two SNP sets were selected (Table 5). Among the selected SNPs, we found no SNPs that were reported in previous studies. However, in the first SNP set, *ATP10B*, *MACROD2*, *and HIP2*, to which *rs6893893*, *rs6079272*, *and rs4518599* respectively annotated, were reported to associate with various BMI-related traits [39–41]. Moreover, in the second SNP set, *ZCCHC17*, the location of *rs6656287*, was previous associated with alcohol dependence, which may affect eating behavior [42,43].

**Table 5:** KARE BMI data analysis result (Entrance cutoff = 5×10^−5^, Removal cutoff = 5×10^−5^).

Set	SNP name	GENE
1^st^ Set	*rs6893893*,*rs2196534*,*rs6079272*, *rs1736913*,*rs4639483*,*rs6462517*, *rs4518599*,*rs10878690*, *rs1012780*, *rs7107562*,*rs11682163*	*ATP10B*, *Unknown*, *MACROD2*, *HLA-F-AS1*, *RSPO2*, *AK025321*, *HIP1*, *AK055974*, *LOC100289473*, *Unknown*, *ALLC*
2^nd^ Set	*rs6656287*	*ZCCHC17*

## Conclusions

Our stepwise CMH method has two large advantages over stepwise logistic regression. The first is that it addresses the sparsity problem, as variance inflation can only be induced in the presence of sparse cells of a genotype count table. Secondly, while logistic regression suffers from intensive computing time (necessary for its iterative optimization algorithm), the stepwise CMH test avoids this problem, as the CMH test statistic is calculated by a simple matrix operation, and the standard error is not affected by the sparsity of cells. In GWAS, as the number of SNPs increases, the chance of including rare SNPs in the stepwise procedure also increases, making it difficult for logistic regression to identify high-order joint identification. Therefore, the stepwise CMH approach is a more appropriate approach than stepwise logistic regression for identification of rare variants in GWAS.

Even though the CMH statistic was originally proposed for detecting conditional independence, a specific SNP set identified via the stepwise CMH approach is informative for identifying joint genetic variants, as the forward and backward steps guarantee that all of the components in the SNP set are significant in the presence of the other SNPs.

Recently, many variable selection methods were developed which use penalization such as LASSO or SCAD [44,45]. We have not directly compared the proposed CMH method to these penalized approaches, due to the fact that our stepwise CMH method tends to select a small number of SNPs with joint effects, while the penalized approaches tend to select a large number of SNPs, if an optimal value of tuning parameter is selected via cross validation. In future comparative studies, we will compare our stepwise CMH to the penalization approaches, while also controlling the number of variables selected. In the presence of ordinal or multinomial traits [46,47], we expect the usefulness of our approach to increase.

Our method focuses on statistical analysis of common variants from GWAS. The traditional GWAS are usually based on the assumption of common disease and common variant (CD-CV). A next generation sequencing (NGS) technique adopts the assumption of common disease and rare variant (CD-RV). Recently, several gene-based aggregation methods for the analysis of rare variants have been proposed [48, 49]. A more complete review of aggregation methods, please refer to [50, 51]. Those aggregation methods are powerful in detecting causal rare variants which are expected to explain missing heritability. However, they may have low power when only a small portion of variants are causal in a region [52]. We are working on developing the stepwise CMH type of statistics for the rare variant analysis.

R code for the stepwise CMH test is provided at a dedicated website (http://bibs.snu.ac.kr/software/stepCMH).
